# Effect of nitrogen-rich cell culture surfaces on type X collagen expression by bovine growth plate chondrocytes

**DOI:** 10.1186/1475-925X-10-4

**Published:** 2011-01-18

**Authors:** Alain Petit, Caroline N Demers, Pierre-Luc Girard-Lauriault, Dorothy Stachura, Michael R Wertheimer, John Antoniou, Fackson Mwale

**Affiliations:** 1 Lady Davis Institute for Medical Research, SMBD-Jewish General Hospital, 3755 Chemin de la Cote Ste-Catherine, Montreal, QC H3T 1E2, Canada; 2 Division of Orthopaedic Surgery, McGill University Health Centre, 1650 Cedar Avenue, Montreal, QC H3G 1A4, Canada; 3 Department of Engineering Physics, École Polytechnique, 2500, Chemin de Polytechnique, Montréal, QC H3T 1J4, Canada

## Abstract

**Background:**

Recent evidence indicates that osteoarthritis (OA) may be a systemic disease since mesenchymal stem cells (MSCs) from OA patients express type X collagen, a marker of late stage chondrocyte hypertrophy (associated with endochondral ossification). We recently showed that the expression of type X collagen was suppressed when MSCs from OA patients were cultured on nitrogen (N)-rich plasma polymer layers, which we call "PPE:N" (N-doped plasma-polymerized ethylene, containing up to 36 atomic percentage (at.% ) of N.

**Methods:**

In the present study, we examined the expression of type X collagen in fetal bovine growth plate chondrocytes (containing hypertrophic chondrocytes) cultured on PPE:N. We also studied the effect of PPE:N on the expression of matrix molecules such as type II collagen and aggrecan, as well as on proteases (matrix metalloproteinase-13 (MMP-13) and molecules implicated in cell division (cyclin B2). Two other culture surfaces, "hydrophilic" polystyrene (PS, regular culture dishes) and nitrogen-containing cation polystyrene (Primaria^®^), were also investigated for comparison.

**Results:**

Results showed that type X collagen mRNA levels were suppressed when cultured for 4 days on PPE:N, suggesting that type X collagen is regulated similarly in hypertrophic chondrocytes and in human MSCs from OA patients. However, the levels of type X collagen mRNA almost returned to control value after 20 days in culture on these surfaces. Culture on the various surfaces had no significant effects on type II collagen, aggrecan, MMP-13, and cyclin B2 mRNA levels.

**Conclusion:**

Hypertrophy is diminished by culturing growth plate chondrocytes on nitrogen-rich surfaces, a mechanism that is beneficial for MSC chondrogenesis. Furthermore, one major advantage of such "intelligent surfaces" over recombinant growth factors for tissue engineering and cartilage repair is potentially large cost-saving.

## Background

Endochondral ossification involves the expression of type X collagen, a marker of chondrocyte hypertrophy [1-3]. Recent evidence indicates that a major drawback of current cartilage- and disc-tissue engineering is that human mesenchymal stem cells (MSCs) from osteoarthritic (OA) patients express type X collagen [4]. We have shown that synthetic polymer surfaces created by glow discharge plasma can suppress the expression of genes associated with hypertrophy in committed human MSCs from OA patients [5-7]. However, little is known about the effect of different culture surfaces on gene expression in the case of growth plate chondrocytes.

Endochondral ossification begins during long bone formation in the embryo [8]. After birth, until adulthood, growth of the long bone is centred in the cartilagenous growth plates, leading to an increase in bone length and epiphyseal growth. It is also an essential component of fracture repair. The primary mammalian growth plate can be divided into several zones, namely the resting, proliferative, and hypertrophic zones [8]. The resting zone chondrocytes elaborate an extracellular matrix similar to the proliferating zone cells, one which expresses type II collagen and the proteoglycan aggrecan; these constitute, together with other matrix molecules, an extensive extracellular matrix. In the proliferative zone, chondrocytes divide actively and synthesize different collagen molecules (types II, IX, and XI) and cartilage-specific proteoglycans [1,2,8]. At this point in time, they express cell cycle-related genes such as cyclins [8]. After cessation of cell division, chondrocytes partly resorb their extracellular matrix and enlarge (become hypertrophic) as they express type X collagen. The up-regulation of type X collagen expression signals the change in chondrocytic phenotype from prehypertrophic to hypertrophic, after which the matrix of the longitudinal septa between the cells starts to mineralize [2,8]. This coordinated proliferation and differentiation of growth plate chondrocytes is required for normal growth and development of the skeleton [9-14].

We recently showed that a novel atmospheric-pressure plasma-polymerized thin film material, named "*nitrogen-rich plasma-polymerized ethylene*" (PPE:N), is able to inhibit hypertrophy as well as osteogenesis in committed human MSCs from OA patients [6]. In contrast, neither aggrecan nor type I collagen expression were significantly affected. These results indicated that PPE:N coatings may be suitable surfaces for inducing MSCs to a chondrocyte or disc-like (nucleus pulposus) phenotype for tissue engineering of cartilage or intervertebral discs, respectively, in which hypertrophy and osteogenesis must be avoided.

In this study, the effect of culturing growth plate chondrocytes expressing the hypertrophic phenotype (cells that express type X collagen) on PPE:N, Primaria^®^, or regular polystyrene (PS) culture dishes was investigated using reverse transcriptase (RT) and polymerase chain reaction (PCR). Primaria^® ^was chosen because it has been described as having nitrogen-containing cations at its surface [15,16]. Thus, we set out to test the hypothesis that the chemically-bound nitrogen content, [N], may be an important regulator of cellular hypertrophy. We demonstrated that, similarly to what we observed in human MSCs, fetal bovine growth plate hypertrophic chondrocytes respond to N-rich substrates by down-regulation of type X collagen expression. This may be important in designing substrates for cartilage- or intervertebral disc repair, where prevention of hypertrophy and endochondral ossification is important. Our findings reveal hitherto unsuspected similarities in regulation of expression of type X collagen in these cell types, and they may provide novel insights into how these cells interact with PPE:N surfaces.

## Methods

### A. Deposition of PPE:N

The methods employed have been described earlier by Girard-Lauriault *et al.*[17,18]. for the experiments reported here, PPE:N films were deposited on biaxially oriented polypropylene (BOPP; 3M Company) [6,17-20]. Using this method, films containing 30 at.% nitrogen, [N], were deposited from the precursor gas mixture composed of nitrogen (N_2_, 10 standard liters per minute, slm) and ethylene (C_2_H_4_, 10 standard cubic centimeters per minute, sccm), the only mixture used in this particular study, unlike our above-referenced earlier work [6,17,18].

To visualize the role of nitrogen content on the attachment of chondrocytes to PPE:N surfaces, micropatterning experiments were carried out as follows: Special 25 μm thick Kapton^® ^polyimide masks were placed over the BOPP substrate. These masks were fabricated with an excimer laser, coupled to a precise positioning system, to create square arrays of holes (diameter: 100 μm; pitch: 200 μm) on an area of 4 cm^2^. Care was taken to assure intimate contact between the mask and substrate surface during deposition runs.

### B. Surface characterization

The surface compositions of the different cell culture surfaces (PPE:N as well as polystyrene (PS) and Primaria^®^; BD Biosciences, Mississauga, ON) were determined by X-ray photoelectron spectroscopy (XPS) [6,17-20]. Throughout this article, we will be referring to their surface elemental concentrations, [X], in terms of the elements that comprise them, namely N, C and O; since hydrogen cannot be detected by XPS, [X] is given by:

$$\left[X\right]=\frac{X}{N+C+O}\times 100\%$$

N, O, and C being determined from the XPS broad-scan spectra.

### C. Growth plate chondrocyte isolation

The physes of multiple primary growth plates were isolated from bovine fetuses, as described earlier [1]. These slices (~2 mm thick) were predominantly from hypertrophic zones. Fetal age, on average 171 days (range 154 - 216 days), was determined by measurement of tibial length [21]. Slices were held for up to 2 h at room temperature in Dulbecco's modified Eagle's medium (DMEM), pH 7.4, containing (per ml) 100 U penicillin, 100 μg streptomycin (medium A) supplemented with 0.25 μg amphotericin B, prior to chondrocyte isolation. The growth plates were digested for 12 to 16 h using collagenase (0.7% w/v) and hyaluronidase (0.2% w/v) in the presence of medium A, supplemented with 10% fetal bovine serum (FBS; Hyclone, Logan, UT), as described previously [1].

### D. Cell culture

After isolation, these heterogeneous chondrocytes expressed type X collagen, a definitive marker of the hypertrophic phenotype (see Results section). Cells were counted with a hemacytometer and 1 million cells in 40 μl of medium A, supplemented with 5 μg/ml insulin, 5 μg/ml transferrin, 5 ng/ml sodium selenite, 1 mg/ml bovine serum albumin, 60 μg/ml ß-glycerophosphate, and freshly prepared 50 μg/ml ascorbic acid, were carefully pipetted onto the centers of the PPE:N surfaces (covering the entire surface of the wells), Primaria^® ^24-well culture dishes (BD Biosciences), or regular 24-well PS culture dishes. The cells were left to adhere to the surfaces for 1 h. Medium was carefully removed and 2 ml of fresh medium was added. Bovine chondrocytes adhered and grew on the three surfaces. Media was changed every 2 days, up to 20 days in culture.

### E. Total RNA isolation

Total RNA was extracted from chondrocytes by a modification of the method of Chomcynski and Sacchi [22] using TRIzol reagent (Invitrogen, Burlington, ON). After centrifugation for 15 min at 12,000 × *g *at 4°C, the aqueous phase was precipitated in 0.5 volume of isopropanol, incubated for 10 min at room temperature, and centrifuged again for 10 min at 12,000 × *g *at 4°C. The resulting RNA pellet was washed in 75% ethanol, centrifuged for 5 min at 7,500 × g at 4°C, air-dried, resuspended in 25 μl of diethylpyrocarbonate-treated water, and assayed for RNA concentration and purity by measuring *A*_260_/*A*_280_.

### F. Reverse transcriptase (RT) and polymerase chain reaction (PCR)

The RT reaction was performed using 0.2 μg total RNA isolated from the chondrocytes in a total volume of 20 μ1, containing 50 mM Tris-HCl (pH 8.3), 75 mM KCl, 3 mM MgCl_2_, 10 mM DTT, 0.5 mM each dATP, dGTP, dCTP and dTTP, and 200 units of Superscript II™ RNAse H^-^reverse transcriptase (Invitrogen).

PCR was performed in a total volume of 25 μ1 containing: 20 mM Tris-HCl (pH 8.4), 50 mM KCl, 1.5 mM MgCl_2 _, 0.2 mM of dATP, dGTP, dCTP, dTTP, 0.8 μM of each primer, 1 μl of RT mixture and 1.25 units of Taq DNA polymerase (Invitrogen). The 30 cycles of PCR included denaturation (95°C, 1 min), annealing (50°C, 60 sec) and extension (72°C, 5 min), as previously described [5-7]. After agarose (2%) gel electrophoresis, PCR products were visualized by ethidium bromide staining and analyzed using a Bio-Rad VersaDoc image analysis system, equipped with a cooled 12-bit CCD camera (Bio-Rad, Mississauga ON). The intensity of the bands was quantified using Quantity One software on the VersaDoc. 18S rRNA level was used as housekeeping gene and served to normalized the results. The primer sequences used for PCR, shown in Table 1, were chosen because they are specific for bovine mRNA and they amplify a single product.

**Table 1:** Primer sequences used for PCR

*Genes*	*Primers*	*Size (bp)*
Aggrecan	5-CAGAACATGCGCTCCAATGA-3' 5-CGTCATAGGTTTCGTTGGTG-3'	371
Type II collagen	5-AACCCAGAAACAACACAATCC-3' 5'-GAGGGGAGAAAAGTCCGAAC-3'	168
Type X collagen	5'-CTGAGCGATACCAAACACC-3' 5'-CCTCTCAGTGATACACCTTTAC-3'	128
MMP-13 Cyclin B2	5'-GATAAAGACTATCCGAGAC-3' 5'-CGAACAATACGGTTACTC-3' 5'-GTTGACTATGACATGGTG-3' 5'-CAAGACAAAGTGCACGAAC-3'	168 358
18S rRNA	5'-CTACTTGGATAACTGTGGTAATTC-3' 5'-GACTCTAGATAACCTCG-3'	168

### G. Statistical Analysis

The non-parametric Kruskal-Wallis test was performed to test for differences between surfaces for each gene, and for changes between the different days of culture. Results were considered significant for p < 0.05.

## Results

### A. Characteristics of polystyrene, Primaria^®^, and PPE:N coatings

**Figure 1:**
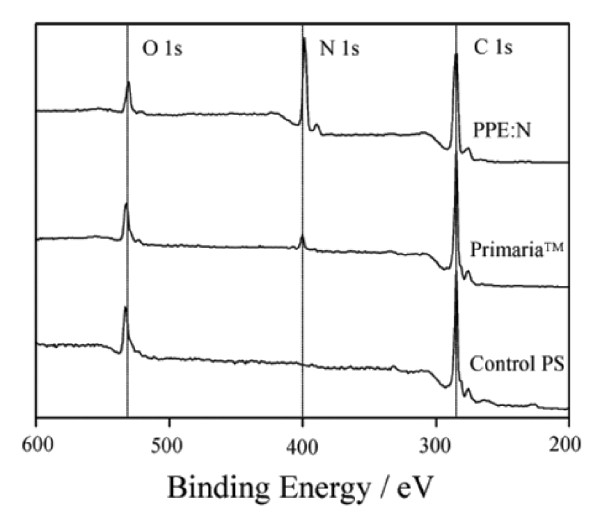
**XPS broad-scan spectra of commercial polystyrene and Primaria^® ^culture dishes, and of PPE:N surfaces**.

X-ray photoelectron spectroscopy (XPS) broad-scan spectra of the different surfaces show peaks characteristic of N, O, and C (Figure 1). The concentrations of elements are expressed as atomic (at.) percentages (%); those of N were 0%, 6%, and 29.5% (all at. %) for PS, Primaria^®^, and PPE:N, respectively (Table 2). The respective concentration of O were 18%, 15%, and 5%, while those of C were 82%, 79%, and 65.5% for PS, Primaria^®^, and PPE:N, respectively, again all in at.%.

**Table 2:** Elemental compositions from XPS analyses of the polystyrene, Primaria^®^, and PPE:N surfaces

*Culture Surfaces*	*N (at. %)*	*O (at. %)*	*C (at. %)*
Control polystyrene	0	18	82
Primaria^®^	6	15	79
**PPE:N**	29.5	5	65.5

Note: Hydrogen cannot be detected by XPS.

### B. Micropatterning

**Figure 2:**
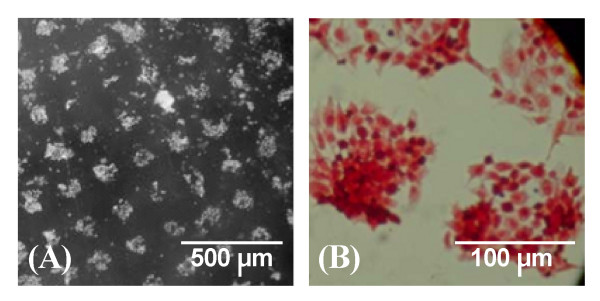
**Micro-patterning of growth plate chondrocytes on a PPE:N-coated surface**. Kapton^® ^polyimide masks (100 μm openings, 200 μm pitch) were placed over the BOPP substrate to create the pattern. Growth plate chondrocytes grown on this patterned surface for 9 days and photomicrographs were taken (A). Proteoglycan production was visualized using Safranin-O staining (B).

Figure 2 shows that chondrocytes cultured on micro-patterned surfaces preferentially grow on nitrogen-rich surfaces (Figure 2A), indicating that the nitrogen-containing functional groups (notably primary amines, see further below) may be responsible for inducing the attachment of chondrocytes. However, the surface of cell attachment exceeded the mask hole (100 μm) in some places, indicating that chondrocytes also grew on the BOPP substrate, but to a lesser extent, or that contact between the mask and substrate surface was not perfectly intimate and that some functional nitrogen groups were formed around the hole. Results also showed that cells that adhere to nitrogen surfaces expressed proteoglycans, as visualized by Safranin-O staining (Figure 2B).

### C. Gene expression

**Figure 3:**
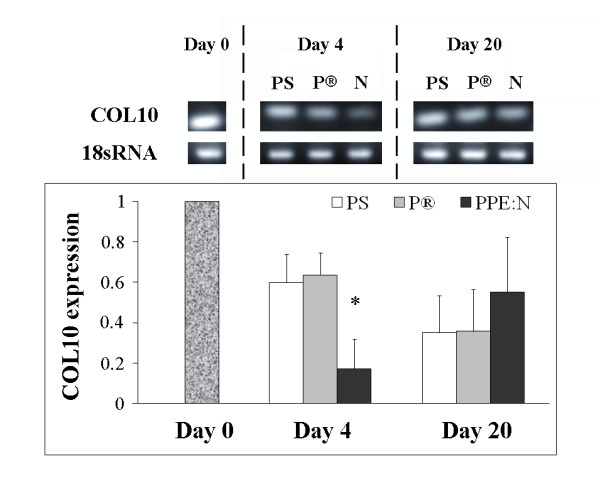
**Effect of polystyrene culture dishes (PS), Primaria^® ^(P), and PPE:N (N) surfaces on the level of type X collagen mRNA in hypertrophic growth plate chondrocytes**. Bovine hypertrophic growth plate chondrocytes were cultured for up to 20 days on the different surfaces and mRNA levels were analyzed by RT-PCR. Agarose gels show representative examples of PCR products for type X collagen (COL 10) mRNA and 18S rRNA. All values were normalized to 18S rRNA. Quantitative results are the mean ± standard error of 4 experiments. * p < 0.05 vs. PS

We recently showed that PPE:N surfaces decreased the expression of type X collagen in MSCs from OA patients. Here, we explored the effect of PPE:N surfaces on the expression of this gene in bovine growth plate chondrocytes. Bovine chondrocytes adhered to the three surfaces compared in the present study and covered the entire surface of the dishes by the end of the culture period (20 days) (results not shown). Figure 3 demonstrates that fetal bovine growth plate chondrocytes isolated without separating the different subpopulations, were enriched with terminally differentiated cells that are characterized by the expression of type X collagen (Day 0), a marker of hypertrophic chondrocytes.

The expression of type X collagen mRNA was significantly lower after 4 days of culture (p = 0.007) when chondrocytes were cultured on PPE:N, compared to control PS and Primaria^®^, suggesting that type X collagen is regulated similarly in hypertrophic chondrocytes and in human MSCs from OA patients (Figure 3). However, and contrary to what was observed in MSCs [5-7], the level of type X collagen mRNA almost returned to control value after 20 days in culture on PPE:N surfaces.

**Figure 4:**
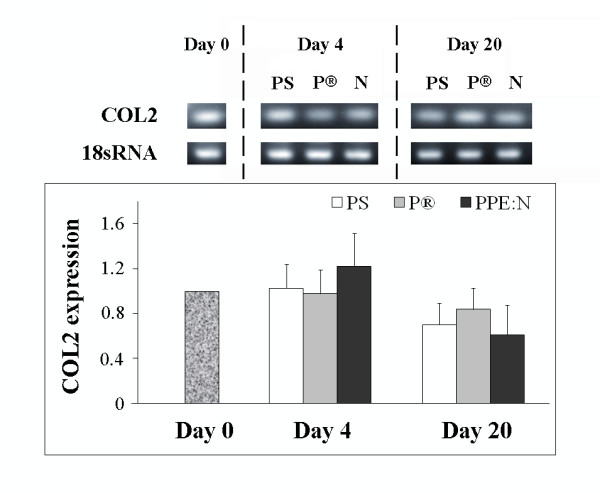
**Effect of polystyrene culture dishes (PS), Primaria^® ^(P), and PPE:N (N) surfaces on the level of type II collagen mRNA in hypertrophic growth plate chondrocytes**. Bovine hypertrophic growth plate chondrocytes were cultured for up to 20 days on the different surfaces and mRNA levels were analyzed by RT-PCR. Agarose gels show representative examples of PCR products for type II collagen (COL 2) mRNA and 18S rRNA. All values were normalized to 18S rRNA. Quantitative results are the mean ± standard error of 4 experiments.

**Figure 5:**
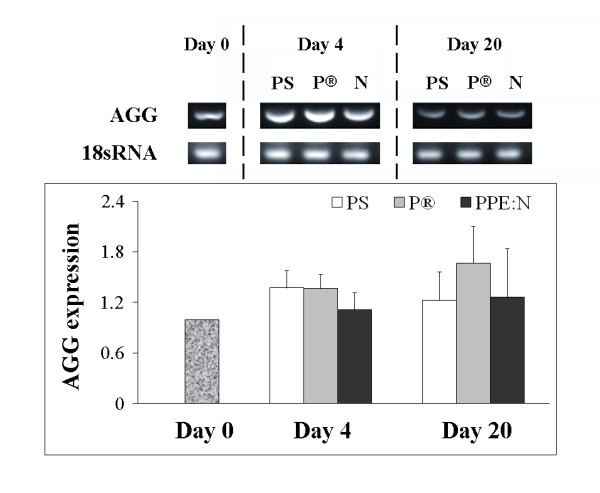
**Effect of polystyrene culture dishes (PS), Primaria^® ^(P), and PPE:N (N) surfaces on the level of aggrecan mRNA in hypertrophic growth plate chondrocytes**. Bovine hypertrophic growth plate chondrocytes were cultured for up to 20 days on the different surfaces and mRNA levels were analyzed by RT-PCR. Agarose gels show representative examples of PCR products for aggrecan (AGG) mRNA and 18S rRNA. All values were normalized to 18S rRNA. Quantitative results are the mean ± standard error of 4 experiments.

Since growth plate chondrocytes secrete an extensive hyaline extracellular matrix consisting principally of type II collagen and the large aggregating proteoglycan aggrecan, we decided to examine the effect of the different culture surfaces on the expression of these genes in growth plate chondrocytes. The levels of type II collagen mRNA did not vary significantly in cells cultured for 4 or 20 days on PS (p > 0.05) (Figure 4): In contrast to type X collagen, type II collagen mRNA levels were not significantly affected by PPE:N surfaces. The different surfaces and times in culture had little effect on levels of aggrecan mRNA (Figure 5).

**Figure 6:**
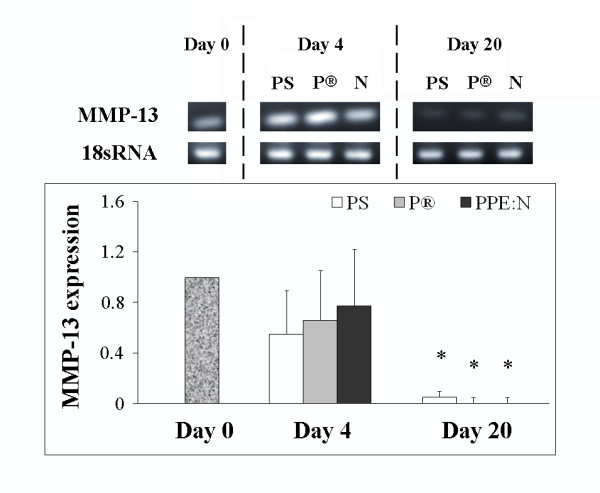
**Effect of polystyrene culture dishes (PS), Primaria^® ^(P), and PPE:N (N) surfaces on the level of MMP-13 mRNA in hypertrophic growth plate chondrocytes**. Bovine hypertrophic growth plate chondrocytes were cultured for up to 20 days on the different surfaces and mRNA levels were analyzed by RT-PCR. Agarose gels show representative examples of PCR products for MMP-13 mRNA and 18S rRNA. All values were normalized to 18S rRNA. Quantitative results are the mean ± standard error of 4 experiments. * p < 0.05 vs. day 4

Collagenase 3 (MMP-13) was examined because it is the most important collagenase found in the growth plate [23], although MMP-16 is also present. We have shown that MMP-13 is up-regulated during chondrocyte hypertrophy in the growth plate [1,2]. Therefore, we next compared the expression of MMP-13 in growth plate chondrocytes cultured on the different surfaces (Figure 6). MMP-13 mRNA levels were expressed maximally on day 4 and declined significantly on day 20 (p = 0.04). However, there were no noteworthy differences in its levels in cells cultured on the three different surfaces.

**Figure 7:**
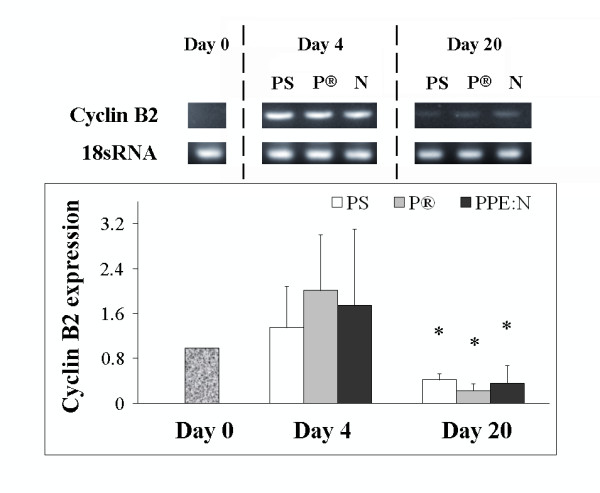
**Effect of polystyrene culture dishes (PS), Primaria^® ^(P), and PPE:N (N) surfaces on the level of cyclin B2 mRNA in hypertrophic growth plate chondrocytes**. Bovine hypertrophic growth plate chondrocytes were cultured for up to 20 days on the different surfaces and mRNA levels were analyzed by RT-PCR. Agarose gels show representative examples of PCR products for cyclin B2 mRNA and 18S rRNA. All values were normalized to 18S rRNA. Quantitative results are the mean ± standard error of 4 experiments. *p < 0.05 vs. day 4

Since the upregulation of MMP-13 expression is observed immediately before and at the onset of cell division, as defined by cyclin B2 expression, and again in chondrocytes that undergo hypertrophy [3], we next analyzed the effect of the different culture surfaces on cyclin B2 mRNA levels (Figure 7). Cyclin B2 mRNA was also expressed maximally on day 4 and declined significantly on day 20. Here too, there were no appreciable differences in its levels in growth plate chondrocytes cells cultured on the three different surfaces.

## Discussion

The role of cell-biomaterial interactions in tissue engineering is still quite poorly understood. It has been known for some time that cells may be sensitive to subtle differences in surface chemistry [5-7,15,16,24-31]. The chemical and topographical nature of the surface can directly influence cellular responses [5,15,16,18,25,29-32], ultimately affecting the rate and quality of new tissue formation [25,33,34]. Our previous studies have shown that PPE:N suppresses the expression not only of type X collagen in MSCs from OA patients, but also of osteogenic marker genes such as alkaline phosphatase, bone sialoprotein, and osteocalcin [6]. In contrast, neither aggrecan nor type I collagen expression were found to be significantly affected. Furthermore, plasma-modified polypropylene or nylon-6 was found to affect type X collagen expression in human MSCs from OA patients [5,7].

Recent studies have attempted to use growth factors to inhibit type X collagen expression [35]. However, no earlier study had so far addressed the possible effect of the substratum on growth plate chondrocyte hypertrophy. The recent advances in our group in creating novel bioactive synthetic polymer surfaces with the aid of ultraviolet-photochemical and plasma-chemical processes [5,17-19,29], particularly the latter, have enabled us to study the culture of MSCs on nitrogen-enriched (nitrogen concentration, [N], up to ~20%) surface-modified polymers such as BOPP and Nylon-6 polyamide [5,7], as well as on super-rich ([N] ≥ 25%) plasma-polymerized thin films, PPE:N [6,17,18]. The latter substrates have distinguished themselves by their ability to adhere certain cell types that earlier resisted adhesion to all prior-known cell culture surfaces, for example, human U937 monocytes [17,18,29].

The present data indicate that surfaces with high [N] values are capable of suppressing type X collagen mRNA in growth plate chondrocytes, with no significant effects on type II collagen, aggrecan, MMP-13 and cyclin B2 mRNA levels. The similarity in type X collagen suppression by PPE:N in hypertrophic growth plate chondrocytes and in human MSCs from OA patients raises the question of whether similar mechanisms are involved. However, contrary to what was observed in MSCs in which PPE:N reduced type X collagen for long time in culture [6], PPE:N was found to decrease the expression of type X collagen in growth plate chondrocytes only in the short-term. In fact, the decreased type X collagen expression was not observed after 12 days of culture (results not shown). This suggests that the decrease may be associated with the initial preferential adhesion of growth plate chondrocytes, as illustrated in Figure 2. This, in turn, suggests that the effect of PPE:N may vary with the cell type. It is also possible that that the surfaces were altered by chondrocyte metabolism and lost their initial composition. This remains to be investigated. Nevertheless, we now know that the substrates' effect on adhering cells is mediated not by the absolute value of [N], but rather by the concentrations of various chemical functionalities at the surface, for example primary amines, imines, nitriles, amides [18,29]. However, hydroxyls (alcohols) and carboxylic acid groups can also play a role since bound oxygen is always incorporated in plasma polymer films due to the reaction of residual surface radicals with air. In the case of PS or Primaria^®^, bound oxygen is due to plasma-modification of the polystyrene in an O_2_-containing gas mixture. As we recently reported elsewhere [18,29], we also know that primary amines account for 5 to 10% of [N]. We also know that nitriles (-C≡N) also constitute an important surface functionality of PPE:N coatings. Moreover, primary amines are the dominant functionality in the remarkable adhesion behaviour we observed in the case of the U937 macrophages [29]. As a next step in the study of growth plate chondrocyte response on high-[N] culture surfaces, those earlier studies suggest working with coatings prepared by two other techniques in our laboratories, low-pressure plasma polymerisation [36], and vacuum ultraviolet photo-polymerisation [37], both capable of yielding a higher amount of primary amine than those found in the high-pressure plasma polymerised coatings used in this present work.

Finally, although gene expression data may suggest an influence of the nitrogen-rich surfaces on the hypertrophic phenotype, mineralization was not addressed in the present study. It is commonly believed that type X collagen is involved in controlling the later stages of endochondral bone formation [1-3]. In this case, culturing chondrocytes or MSCs on these surfaces should suppress mineralization. This is of great importance for cartilage repair or tissue engineering using MSCs from osteoarthritc patients known to express type X collagen. Further studies are therefore necessary to determine the effect of these surfaces on mineralization.

## Conclusions

Type X collagen expression was reduced, at least transiently, by nitrogen rich surfaces in both growth plate chondrocytes as well as in MSCs from OA patients, which is beneficial to chondrogenesis. As "intelligent" surfaces, PPE:N coatings therefore represent a potentially advantageous cell-culture substrate with beneficial effects for cartilage and intervertebral disc repair. However, further studies are necessary to better understand the nature of specific functional groups, such as primary amines, on gene expression and cell phenotype: this information will be important in tissue engineering applications that require the use of such "intelligent" surfaces.

## Abbreviations

BOPP: biaxially oriented polypropylene; C_2_H_4: _ethylene; DMEM: Dulbecco's modified Eagle's medium; FBS: Fetal bovine serum; MMP: matrix metalloproteinase; MSC: Mesenchymal stem cell; N: Nitrogen; OA: Osteoarthritis; PPPE:N: N-doped plasma-polymerized ethylene; PS: Polystyrene; RT-PCR: Reverse transcriptase-Polymerase chain reaction; SCCM: standard cubic centimeters per minute; SLM: standard liters per minute; XPS: X-ray photoelectron spectroscopy

## Competing interests

The authors declare that they have no competing interests.

## Authors' contributions

AP participated in the interpretation of the results, drafted the Introduction and the Discussion with FM, and revised the final version of the article. CND carried out experiments with stem cells, participated to RT-PCR experiments, and drafted the section on stem cell isolation and culture. PLGL prepared the surfaces and helped to draft the method section on surface preparation. DS participated to cell isolation and RT-PCR analysis. MRW critically revised the manuscript. JA participated in the design of the study and performed the statistical analysis. FW conceived the study and drafted the Introduction and the Discussion with AP. All authors read and approved the final manuscript.

## References

[B1] Mwale F Billinghurst C Wu W Alini M Webber C Reiner A Ionescu M Poole J Poole AR Selective assembly and remodelling of collagens II and IX associated with expression of the chondrocyte hypertrophic phenotype Dev Dyn 2000 218 648 662 10.1002/1097-0177(200008)218:4<648::AID-DVDY1022>3.0.CO;2-P 10906783

[B2] Mwale F Tchetina E Wu CW Poole AR The assembly and remodeling of the extracellular matrix in the growth plate in relationship to mineral deposition and cellular hypertrophy: an in situ study of collagens II and IX and proteoglycan J Bone Miner Res 2002 17 275 283 10.1359/jbmr.2002.17.2.275 11811558

[B3] Tchetina E Mwale F Poole AR Distinct phases of coordinated early and late gene expression in growth plate chondrocytes in relationship to cell proliferation, matrix assembly, remodeling, and cell differentiation J Bone Miner Res 2003 18 844 851 10.1359/jbmr.2003.18.5.844 12733723

[B4] Mwale F Stachura D Roughley P Antoniou J Limitations of using aggrecan and type X collagen as markers of chondrogenesis in mesenchymal stem cell differentiation J Orthop Res 2006 24 1791 1798 10.1002/jor.20200 16779832

[B5] Nelea V Luo L Demers CN Antoniou J Petit A Lerouge S Wertheimer MR Mwale F Selective inhibition of type X collagen expression in human mesenchymal stem cell differentiation on polymer substrates surface-modified by glow discharge plasma J Biomed Mater Res Part A 2005 75A 216 223 10.1002/jbm.a.30402 16044417

[B6] Mwale F Girard-Lauriault PL Wang HT Lerouge S Antoniou J Wertheimer MR Suppression of hypertrophy and osteogenesis in committed human mesenchymal stem cells cultured on novel nitrogen-rich plasma polymer coatings Tissue Eng 2006 12 2639 2647 10.1089/ten.2006.12.2639 16995797

[B7] Mwale F Wang HT Nelea V Luo L Antoniou J Wertheimer MR The effect of glow discharge plasma surface modification of polymers on the osteogenic differentiation of committed human mesenchymal stem cells Biomaterials 2006 27 2258 2264 10.1016/j.biomaterials.2005.11.006 16313952

[B8] Poole AR Laverty S Mwale F Press CU Endochondral bone formation and development in the axial and appendicular skeleton The osteoporosis primer 2000 3 17

[B9] Stanton LA Underhill TM Beier F MAP kinases in chondrocyte differentiation Dev Biol 2003 263 165 715 10.1016/S0012-1606(03)00321-X 14597193

[B10] Wang G Woods A Sabari S Pagnotta L Stanton LA Beier F RhoA/ROCK signaling suppresses hypertrophic chondrocyte differentiation J Biol Chem 2004 279 13205 13214 10.1074/jbc.M311427200 14726536

[B11] Stanton LA Sabari S Sampaio AV Underhill TM Beier F p38 MAP kinase signalling is required for hypertrophic chondrocyte differentiation Biochem J 2004 378 53 62 10.1042/BJ20030874 14594450 PMC1223932

[B12] LuValle P Beier F Cell cycle control in growth plate chondrocytes Front Biosci 2000 5 D493 D503 10.2741/LuValle 10799356

[B13] Beier F Taylor AC LuValle P Activating transcription factor 2 is necessary for maximal activity and serum induction of the cyclin A promoter in chondrocytes J Biol Chem 2000 275 12948 12953 10.1074/jbc.275.17.12948 10777595

[B14] Beier F Leask TA Haque S Chow C Taylor AC Lee RJ Pestell RG Ballock RT LuValle P Cell cycle genes in chondrocyte proliferation and differentiation Matrix Biol 1999 18 109 120 10.1016/S0945-053X(99)00009-8 10372550

[B15] Klein-Soyer C Hemmendinger S Cazenave J-P Culture of human vascular endothelial cells on a positively charged polystyrene surface, Primaria: comparison with fibronectin-coated tissue culture grade polystyrene Biomaterials 1989 10 85 90 10.1016/0142-9612(89)90036-7 2706306

[B16] Steele JG Dalton BA Johnson G Underwood PA Adsorption of fibronectin and vitronectin onto Primaria^® ^and tissue culture polystyrene and relationship to the mechanism of initial attachment of human vein endothelial cells and BHK-21 fibroblasts Biomaterials 1995 16 1057 1067 10.1016/0142-9612(95)98901-P 8519926

[B17] Girard-Lauriault P-L Mwale F Iordanova M Demers CN Desjardins P Wertheimer MR Atmospheric pressure deposition of micropatterned N-rich plasma-polymer films for tissue engineering Plasma Process Polym 2005 2 263 270 10.1002/ppap.200400092

[B18] Girard-Lauriault PL Desjardins P Unger WES Lippitz A Wertheimer MR Chemical characterisation of nitrogen-rich plasma-polymer films deposited in dielectric barrier discharges at atmospheric pressure Plasma Process Polym 2008 5 631 644 10.1002/ppap.200800054

[B19] Bullett NA Bullett DP Truica-Marasecu F Lerouge S Mwale F Wertheimer MR Polymer surface micropatterning by plasma and VUV-photochemical modification for controlled cell culture Appl Surf Sci 2004 235 395 405 10.1016/j.apsusc.2004.02.058

[B20] Guimond S Radu I Czeremuszkin G Carlsson DJ Wertheimer MR Biaxially oriented polypropylene (BOPP) surface modification by nitrogen atmospheric pressure glow discharge (APGD) and by air corona Plasmas Polym 2002 7 71 88 10.1023/A:1015274118642

[B21] Pal S Tang LH Choi H Habermann E Rosenberg L Roughley P Poole AR Structural changes during development in bovine fetal epiphyseal cartilage Coll Relat Res 1981 1 151 176 7346224 10.1016/s0174-173x(81)80017-9

[B22] Chomczynski P Sacchi N Single-step method of RNA isolation by acid guanidinium thiocyanate-phenol-chloroform extraction Anal Biochem 1987 162 156 159 10.1016/0003-2697(87)90021-2 2440339

[B23] Wu CW Tchetina EV Mwale F Hasty K Pidoux I Reiner A Chen J Van W Poole AR Proteolysis involving matrix metalloproteinase 13 (collagenase-3) is required for chondrocyte differentiation that is associated with matrix mineralization J Bone Miner Res 2002 17 639 651 10.1359/jbmr.2002.17.4.639 11918221

[B24] Acarturk TO Peel MM Petrosko P LaFramboise W Johnson PC DiMilla PA Control of attachment, morphology, and proliferation of skeletal myoblasts on silanized glass J Biomed Mater Res 1999 44 355 370 10.1002/(SICI)1097-4636(19990315)44:4<355::AID-JBM1>3.0.CO;2-B 10397939

[B25] Boyan BD Hummert TW Dean DD Schwartz Z Role of material surfaces in regulating bone and cartilage cell response Biomaterials 1996 17 137 146 10.1016/0142-9612(96)85758-9 8624390

[B26] Britland S Clark P Connolly P Moores G Micropatterned substratum adhesiveness: a model for morphogenetic cues controlling cell behavior Exp Cell Res 1992 198 124 129 10.1016/0014-4827(92)90157-4 1727046

[B27] Hu Y Winn SR Krajbich I Hollinger O Porous polymer scaffolds surface-modified with arginine-glycine-aspartic acid enhance bone cell attachment and differentiation in vitro J Biomed Mater Res Part A 2003 64A 583 590 10.1002/jbm.a.10438 12579573

[B28] Curran JM Chen R Hunt JA Controlling the phenotype and function of mesenchymal stem cells in vitro by adhesion to silane-modified clean glass surfaces Biomaterials 2005 26 7057 7067 10.1016/j.biomaterials.2005.05.008 16023712

[B29] Girard-Lauriault P-L Truica-Marasescu F Petit A Wang HT Desjardins P Antoniou J Mwale F Wertheimer MR Adhesion of human U937 monocytes to nitrogen-rich organic thin films: novel insights into the mechanism of cellular adhesion Macromol Biosci 2009 9 911 921 10.1002/mabi.200800359 19472170

[B30] Mwale F Petit A Wang HT Epure LM Girard-Lauriault P-L Ouellet JA Wertheimer MR Antoniou J The potential of N-rich plasma-polymerized ethylene (PPE:N) films for regulating the phenotype of the nucleus pulposus The Open Orthop J 2008 2 137 144 10.2174/1874325000802010137 PMC2687122 19478889

[B31] Mwale F Wang HT Petit A Girard-Lauriault P-L Hunter CJ Ouellet JA Wertheimer MR Antoniou J The effect of novel nitrogen-rich plasma polymer coatings on the phenotypic profile of notochordal cells Biomed Eng Online 2007 6 33 10.1186/1475-925X-6-33 17822560 PMC2018722

[B32] Salasznyk RM Williams WA Boskey A Batorsky A Plopper GE Adhesion to vitronectin and collagen I promotes osteogenic differentiation of human mesenchymal stem cells J Biomed Biotechnol 2004 2004 24 34 10.1155/S1110724304306017 15123885 PMC545655

[B33] Li WJ Tuli R Huang X Laquerriere P Tuan RS Multilineage differentiation of human mesenchymal stem cells in a three-dimensional nanofibrous scaffold Biomaterials 2005 26 5158 5166 10.1016/j.biomaterials.2005.01.002 15792543

[B34] Mwale F Iordanova M Demers CN Steffen T Roughley P Antoniou J Biological evaluation of chitosan salts cross-linked to genipin as a cell scaffold for disk tissue engineering Tissue Eng 2005 11 130 140 10.1089/ten.2005.11.130 15738668

[B35] Steinert A Weber M Dimmler A Julius C Schutze N Noth U Cramer H Eulert J Zimmermann U Hendrich C Chondrogenic differentiation of mesenchymal progenitor cells encapsulated in ultrahigh-viscosity alginate J Orthop Res 2003 21 1090 1097 10.1016/S0736-0266(03)00100-1 14554223

[B36] Truica-Marasescu F-E Wertheimer MR Nitrogen-rich plasma-polymer films for biomedical applications Plasma Process Polym 2008 5 44 57 10.1002/ppap.200700077

[B37] Truica-Marasescu F Wertheimer MR Vacuum ultraviolet (VUV) photo-polymerisation of amine-rich thin films Macromol Chem Phys 2008 209 1043 1049 {Erratum: 2008, 209: 2061}. 10.1002/macp.200800089

